# Screening for PTSD and TBI in Veterans using Routine Clinical Laboratory Blood Tests

**DOI:** 10.1038/s41398-022-02298-x

**Published:** 2023-02-21

**Authors:** Mu Xu, Ziqiang Lin, Carole E. Siegel, Eugene M. Laska, Duna Abu-Amara, Afia Genfi, Jennifer Newman, Michelle K. Jeffers, Esther M. Blessing, Steven R. Flanagan, Silvia Fossati, Amit Etkin, Charles R. Marmar

**Affiliations:** 1 Center for Precision Medicine in Alcohol Use Disorder and PTSD, Department of Psychiatry, New York University Grossman School of Medicine, New York, NY USA; 2 Department of Psychiatry, New York University Grossman School of Medicine, New York, NY USA; 3 Steven and Alexandra Cohen Veteran Center for the Study of PTSD and TBI, Department of Psychiatry, New York University Grossman School of Medicine, New York, NY USA; 4 Department of Population Health, New York University Grossman School of Medicine, New York, NY USA; 5 Rusk Rehabilitation Institute, Department of Rehabilitation Medicine, New York University Grossman School of Medicine, New York, NY USA; 6 Department of Pharmacology, Lewis Katz School of Medicine, Temple University, Philadelphia, PA USA; 7 Alzheimer’s Center at Temple, Lewis Katz School of Medicine, Temple University, Philadelphia, PA USA; 8 Department of Psychiatry and Behavioral Sciences, Stanford University, Stanford, CA USA; 9 Alto Neuroscience, Los Altos, CA USA

**Keywords:** Predictive markers, Psychiatric disorders

## Abstract

Post-traumatic stress disorder (PTSD) is a mental disorder diagnosed by clinical interviews, self-report measures and neuropsychological testing. Traumatic brain injury (TBI) can have neuropsychiatric symptoms similar to PTSD. Diagnosing PTSD and TBI is challenging and more so for providers lacking specialized training facing time pressures in primary care and other general medical settings. Diagnosis relies heavily on patient self-report and patients frequently under-report or over-report their symptoms due to stigma or seeking compensation. We aimed to create objective diagnostic screening tests utilizing Clinical Laboratory Improvement Amendments (CLIA) blood tests available in most clinical settings. CLIA blood test results were ascertained in 475 male veterans with and without PTSD and TBI following warzone exposure in Iraq or Afghanistan. Using random forest (RF) methods, four classification models were derived to predict PTSD and TBI status. CLIA features were selected utilizing a stepwise forward variable selection RF procedure. The AUC, accuracy, sensitivity, and specificity were 0.730, 0.706, 0.659, and 0.715, respectively for differentiating PTSD and healthy controls (HC), 0.704, 0.677, 0.671, and 0.681 for TBI vs. HC, 0.739, 0.742, 0.635, and 0.766 for PTSD comorbid with TBI vs HC, and 0.726, 0.723, 0.636, and 0.747 for PTSD vs. TBI. Comorbid alcohol abuse, major depressive disorder, and BMI are not confounders in these RF models. Markers of glucose metabolism and inflammation are among the most significant CLIA features in our models. Routine CLIA blood tests have the potential for discriminating PTSD and TBI cases from healthy controls and from each other. These findings hold promise for the development of accessible and low-cost biomarker tests as screening measures for PTSD and TBI in primary care and specialty settings.

## Introduction

Post-traumatic stress disorder (PTSD) is defined by the co-occurrence of re-experiencing, avoidance of reminders, negative beliefs and feelings and hyper-arousal symptoms in survivors of traumatic events such as child abuse, rape, torture and war. Veterans who have been deployed to warzones are at high risk of developing PTSD. The lifetime prevalence of warzone-related PTSD in US veterans of the Vietnam War and subsequent conflicts, including the Iraq and Afghanistan Wars, is estimated to be between 10.1% and 30.9% [1, 2].

The diagnosis of PTSD is based on clinical criteria defined by the Diagnostic and Statistical Manual (DSM), which are determined by clinical interviews including the gold standard Clinician Administered PTSD Scale (CAPS)., and patient and clinician measures of symptoms and functioning. While PTSD causes substantial psychological distress and functional impairment, a significant portion of those with PTSD do not seek psychiatric care, likely due to a combination of factors including insufficient awareness, avoidance symptoms and social stigma related to feelings of self-blame, shame and weakness. Many of those seeking care following trauma present in primary care settings rather than specialty mental health settings. Primary care providers, including those in military medicine, often have limited training in the diagnosis of PTSD and limited time to assess their patients’ psychiatric problems. Because of shame, guilt and perceived weakness, warfighters frequently underreport their symptoms, while a minority exaggerate or fabricate their symptoms adding to the challenges for diagnosing PTSD including within specialty mental health settings.

PTSD is not the only and not always the most common neuropsychiatric sequelae of warzone service. Among US military veterans with deployments to Iraq and Afghanistan, 10–20% are estimated to have developed a traumatic brain injury (TBI) following blast exposure and other head injuries, with 75% of these classified by the American Congress of Rehabilitative Medicine criteria as a mild traumatic brain injury (mTBI) [3]. Additionally, TBI is a common comorbidity of veterans with PTSD. TBI results in structural and functional disruptions within the brain. Structural damage occurs through the impact of the trauma, which can include skull fracture, blood vessel damage and more commonly diffuse axonal shearing. These injuries subsequently cause inflammation, breakdown of the blood-brain barrier and eventually cell death. While moderate and severe TBI patients have significant trauma history and usually have neuroimaging changes, individuals with mTBI may or may not experience loss of consciousness and retrograde or anterograde amnesia and usually do not have radiological findings in routine head CT and MRI scans. In addition, veterans with mTBI often share similar symptomology with those experiencing PTSD, such as fatigue, depression, cognitive deficits, insomnia and irritability, complicating diagnosis of both conditions [4].

Development of objective biomarkers is an urgent priority for use in screening for PTSD and TBI in primary care settings and for tailoring treatments. Proteomic and lipidomic studies have identified markers, including serum tau fragments that are altered in individuals with mTBI compared to healthy controls, which show promise as diagnostic screening tests [5–7]. In PTSD, until recently, it was believed that there were no objective biomarkers. However, recent advances in biomarker research, including brain imaging [8], blood-based multiomic features [9], blood markers for immune modulation [10, 11] and voice markers [12] show promise for advancing PTSD diagnostic screening with objective data that is independent of self-report biases. Using a multi-omic approach with comprehensive genomic, epigenomic, metabolomic, proteomic and other high throughput measurements, Dean et al. [9] utilized a three-step down-selection process and developed a diagnostic panel with 28 different biological markers. They reported promising results for discriminating PTSD cases from controls (AUC = 0.80, accuracy 0.77, sensitivity 81%, and specificity 73%). This multiomic panel, developed by a Department of Defense (DoD) supported Systems Biology of PTSD consortium, represents one of the first efforts towards the development of a blood test for PTSD. While our PTSD consortium is working toward FDA approval for this blood test, requirements for further validation studies and the need to develop a commercial platform integrating genomic, metabolomic and proteomic markers currently precludes the use of this test in routine clinical settings.

As an alternative approach in the current study, we utilized CLIA lab tests, which are routine clinical blood tests available in primary and specialty medical practice settings, to determine if these tests have utility in discriminating warzone-related PTSD and mild TBI cases from warzone deployed healthy controls and from each other. A challenge of this approach is that many warfighters and civilians with PTSD and mild TBI develop alcohol use disorder (AUD), major depressive disorder (MDD), and obesity as determined by Body Mass Index (BMI), which can cause laboratory abnormalities that are attributable to these comorbidities rather than PTSD and mild TBI per se [13]. In this report, we present our initial findings suggesting the potential use of routine CLIA labs to screen for PTSD and mTBI, controlling for AUD, MDD, and BMI.

## Materials and methods

### Subject sources

We present findings from our Cohen Veteran Center (CVC) dataset which includes male veterans, recruited at NYU Langone Medical Center and Stanford University, with a history of deployment to either Iraq or Afghanistan. Exclusion criteria included current severe medical conditions unrelated to TBI or PTSD, including cerebrovascular disorders, multiple sclerosis, open head injury, cancer, autoimmune conditions and human immunodeficiency virus and severe psychiatric conditions (i.e., schizophrenia or bipolar disorder, history of psychotic disorder, obsessive-compulsive disorder, drug abuse and suicide and homicide risk).

We present results in a sample of 475 Iraq and Afghanistan veterans who had completed clinical assessments and CLIA labs and met selection criteria for one of four groups: PTSD (*n* = 44), TBI (*n* = 144), PTSD with comorbid TBI (*n* = 52), and warzone-deployed healthy controls (HC; *n* = 235).

### PTSD and TBI diagnosis

PTSD diagnosis was established according to DSM-5 criteria using the Clinician-Administered PTSD Scale-5 (CAPS-5). Briefly, CAPS-5 is a 30-item clinical interview, which provides information on the frequency and severity of PTSD symptoms and functioning used to make current (past month) and lifetime diagnoses of PTSD.

mTBI diagnosis was determined by a history of closed head injury with loss of consciousness (LOC), as determined by The Ohio State University TBI Identification Method–Short Form. The Concussion Symptoms Inventory (CSI) was used for the assessment of post-concussive symptoms.

### Assessing alcohol abuse and depression

Current alcohol abuse and depression were diagnosed by DSM IV criteria utilizing the Structured Clinical Interview for DSM-IV, Non-Patient Version (SCID-IV). A participant was considered positive for current alcohol use disorder if they met SCID-IV criteria for current mild to severe alcohol abuse. A participant was considered positive for current depression if they met SCID –IV criteria for current major depressive disorder.

### Blood samples

Fasting venous blood samples were obtained by venipuncture between 0800 and 0900 am, following an overnight fast. Upon arrival at the clinical site, subjects were asked to rest in a supine position for 30 min prior to venipuncture. Nurses completed blood draws using 21 G butterfly needles. Blood samples were immediately delivered to the clinical lab after the blood draw and analyzed immediately for each participant.

### CLIA lab analysis

Complete blood count with differential, basic metabolic panel, hepatic panel, total cholesterol, triglycerides (TG), high-density lipoprotein (HDL), low-density lipoprotein (LDL), hemoglobin A1c (HbA1c), C-reactive protein (CRP), gamma-glutamyl transferase (GGT) and serum insulin were measured using CLIA-certified lab procedures. Insulin resistance (IR) was assessed using the homeostatic model assessment (HOMA-IR), calculated as (glucose × insulin)/(22.5 × 18) [14]. Diabetes was defined by HbA1c ≥ 6.5 or fasting glucose ≥ 126, whereas pre-diabetes was defined by HbA1c 5.7–6.4 or fasting glucose 100–125.

### Adjusting for imbalance in the sample

Because the dataset was imbalanced for the number of individuals in each clinical group, a decision boundary based on an unadjusted analysis is misleading. SMOTE (Synthetic Minority Oversampling Technique) was used to rebalance the data [15–17]. The SMOTE algorithm creates synthetic data points in the feature space by randomly picking a point on a line between two close neighboring subjects. There are several advantages to using SMOTE to rebalance the dataset, including 1) avoidance of overfitting problems, 2) avoidance of information loss, and 3) simplified data implementation and interpretation.

### Feature selection and prediction

Random Forest, a classification method, is an ensemble method comprised of many independent decision trees. Each tree ends in terminal nodes whose composition determines the local predicted class. The overall RF classification is determined by a “vote” summarizing the individual tree results. RF uses a bootstrap method to select a sample from the dataset for each tree to train the decision tree and uses the left out (out-of-bag, OOB) sample to estimate the prediction error [18]. This method provides a measure of the importance of each feature for prediction by adding a noise term to each feature and examining its consequence via changes in the Gini Index, or overall error. The larger the changes, the more important this feature is to the prediction model.

Stepwise forward variable selection was used to maximize the AUC in each of the models. We ascertained 41 CLIA labs, including metabolic markers (fasting glucose and insulin resistance), inflammatory markers, liver function tests and complete blood count (CBC). These features, along with their mean and standard deviations across the study groups, are presented in Table 1.

**Table 1:** Feature value comparisons for study groups.

Feature	HC *n* = 235 Means (sds)	PTSD *n* = 44 Means (sds)	TBI *n* = 158 Means (sds)	PTSD with TBI *n* = 52 Means (sds)
glucose	81.67 (10.68)	88.93 (54.72)	84.09 (15.4)	81.31 (9.1)
bun	14.45 (3.39)	14.27 (3.26)	14.91 (3.13)	14.67 (3.63)
Na	138.79 (2.44)	139.25 (2.04)	139.37 (2.31)*	139.33 (2.56)
K	4.14 (0.35)	4.17 (0.32)	4.2 (0.32)	4.21 (0.35)
Cl	100.98 (2.1)	100.7 (2.19)	101.06 (2.1)	101.04 (2.19)
CO2	28.28 (2)	28.2 (1.96)	27.91 (2.08)	28.15 (2.4)
Ca	9.19 (0.36)	9.23 (0.36)	9.21 (0.3)	9.25 (0.35)
Total Protein	7.26 (0.55)	7.32 (0.58)#	7.15 (0.44)*	7.15 (0.5)
ALB	4.17 (0.3)	4.21 (0.29)	4.19 (0.27)	4.14 (0.27)
BILITOT	0.72 (0.3)	0.75 (0.26)	0.74 (0.37)	0.63 (0.24)
Alkaline phosphatase	65.11 (17.06)	63.02 (20.4)	62.12 (15.48)	70.46 (19.92)*
AST	27.9 (11.64)	29.89 (12.1)	30.41 (13.61)	32.75 (13.8)*
ALT	39.04 (21.43)	39.89 (19.63)	42.49 (21.07)	44.73 (21.02)
GGT	30.03 (23.91)	31.2 (18.84)	33.13 (29.93)	37.96 (27.99)*
creatinine	0.96 (0.15)	1.03 (0.22)*	0.98 (0.15)	0.97 (0.15)
Cholesterol	172.86 (33.92)	181.8 (36.77)	180.39 (35.17)*	177.23 (34.54)
Triglyceride	93.67 (63.47)	97.25 (82.63)	109.53 (93.53)*	127.06 (98.85)*
HDL	52.58 (12.96)	54.61 (13.13)	51.35 (13.77)	47.77 (11.6)*
LDL	101.6 (29.37)	108.8 (31.1)	107.71 (31.24)*	105.31 (29.86)
WBC	5.68 (1.47)	5.85 (1.63)	5.75 (1.74)	6.35 (2.01)*
RBC	4.96 (0.42)	5.04 (0.43)#	4.88 (0.44)	5.01 (0.41)
hgb	14.7 (1.22)	14.9 (1.13)	14.62 (1.07)	14.84 (1.4)
hct	43.94 (3.49)	44.71 (3.00)#	43.49 (3.06)	44.14 (3.49)
mcv	88.85 (4.94)	89.05 (5.13)	89.44 (4.84)	88.11 (4.22)
mch	29.74 (1.97)	29.69 (2.05)	30.07 (1.89)	29.62 (1.97)
mchc	33.46 (0.94)	33.35 (1.13)	33.63 (1)	33.6 (1.14)
RDW	12.3 (0.92)	12.54 (1.13)#	12.19 (0.9)	12.35 (0.8)
Platelet	228.32 (49.5)	232.34 (57.05)	227.91 (55.72)	234.5 (59.76)
Neut%	54.73 (7.87)	54.67 (8.99)	56.65 (8.71)*	54.67 (8.9)
Lymph%	33.47 (7.13)	32.81 (8.18)	31.53 (7.68)*	33.95 (8.45)
Monos%	8.04 (1.82)	8.37 (2.43)	8.19 (1.94)	7.91 (2.26)
Eosino%	2.97 (2.19)	3.42 (2.14)	2.86 (2.1)	2.84 (1.94)
Baso%	0.77 (0.48)	0.76 (0.53)	0.77 (0.48)	0.64 (0.47)
Neut#	3.16 (1.1)	3.29 (1.33)	3.34 (1.47)	3.57 (1.71)*
Lymph#	1.86 (0.48)	1.85 (0.47)	1.76 (0.54)	2.06 (0.52)*
Monos#	0.45 (0.14)	0.47 (0.15)	0.46 (0.16)	0.49 (0.17)
Eosino#	0.17 (0.16)	0.2 (0.14)	0.16 (0.11)	0.18 (0.14)
Baso#	0.04 (0.03)	0.04 (0.03)	0.04 (0.02)	0.04 (0.02)
insulin	7.49 (6.13)	7.23 (4.37)	8.25 (8.1)	10.06 (13.12)*
CRP	1.52 (1.95)	1.82 (2.62)	1.84 (2.18)	2.05 (2.76)
hbA1c	5.30 (0.43)	5.46 (1.01)	5.39 (0.70)	5.45 (0.45)*
Body Mass Index (BMI) [mean (sd)]	27.60 (4.63)	27.74 (4.92)	28.35 (4.28)	28.60 (3.75)
Pulse [mean (sd)]	62.54 (11.42)	64.73 (12.17)	61.94 (10.70)	64.92 (11.20)
HOMA-IR^a^ [mean (sd)]	1.56 (1.48)	1.78 (2.56)	1.85 (2.56)	2.11 (3.01)
Metabolic Syndrome (MetS)^b^ [n (%)]	6 (3.63%)	1 (3.33%)	5 (4.24%)	5 (11.90%)

*HC* healthy controls, *PTSD* post-traumatic stress disorder, *TBI* traumatic brain injury.

^a^HOMA-IR (insulin resistance) is calculated by (glucose × insulin)/22.5.

^b^Metabolic syndrome is defined by at least 3 of the following 5 criteria: 1) fasting glucose ≥ 110 mg/dL, 2) abdominal obesity, based on waist circumference (WC) of > 40 inch (for men), or WC > 35 (for women), 3) TGs ≥ 150 mg/dL, 4) HDL cholesterol < 40 mg/dL (for men) or HDL < 50 mg/dL (for women), and 5) systolic blood pressure ≥ 130 or diastolic blood pressure ≥ 85 mmHg.

*p < 0.05 for PTSD vs HC, TBI vs HC, or PTSD with TBI vs HC.

#*p* < 0.05 for PTSD vs TBI.

Four RF models were used to discriminate PTSD from HC, mTBI from HC, PTSD comorbid with mTBI from HC and PTSD from mTBI based on the best features identified from the stepwise variable selection processes. AUC, accuracy, sensitivity and specificity were computed for each of the four models. The importance ranking of variables, measured by the mean decrease in the Gini impurity index, were also obtained.

### Selection of cut points for determining sensitivity and specificity

For each of the four models we determined sensitivity (SE) and false positive rates (1-Specifity) rates by selecting to a cut point that maximizes the distance of a point on the ROC curve to the line of chance in which true positive rates equal true negative rates. This distance is known as Youden’s Index. The cut point corresponding to its maximum is considered ideal for representing the diagnostic value inherent in the ROC, in that it produces SE and 1-SP furthest from chance values.

### Confounder analysis

In order to determine whether current comorbid alcohol abuse and current depression are confounders of our prediction models, we performed a confounder analysis. As an example, to test if the current MDD is a confounder, two hypotheses need to be rejected for each marker in the final models:: i) H_0_: Pr(CLIA | MDD) = Pr(CLIA) i.e., the CLIA lab values and MDD are independent and ii) H_0_: Pr(PTSD | CLIA, MDD,) = Pr(PTSD | CLIA) i.e., PTSD given CLIA and MDD is conditionally independent of MDD. To test the first hypothesis, Hoeffding’s D nonparametric test of independence or the Kruskall Wallace test as appropriate was used. To test the conditional independence hypothesis, Random Forests (RF) were individually obtained for Pr(PTSD | MDD, CLIA) and compared to Pr(PTSD | MDD) grown from a RF without the CLIA lab values. A test of equality of these probabilities was performed using a one-sample Wilcoxon rank on the differences in probabilities. A similar analysis was performed for current alcohol abuse and BMI.

## Results

Table 2 summarizes demographic data for the participants. The average ages for PTSD, TBI, TBI with PTSD and HC subjects were 32.51 (SD 5.78), 32.21 (SD 7.75), 33.40 (SD 7.84), and 31.78 (SD 6.19), respectively. There were no significant differences comparing HC with PTSD, TBI, and TBI with comorbid PTSD groups in age, education level, and BMI. However, there were significant differences between HC and TBI with comorbid PTSD subjects in ethnicity, and HOMA-IR, although in neither group did participants meet diagnostic criteria for hyperlipidemia or prediabetes.

**Table 2:** Demographics of veterans who completed CLIA blood tests.

	HC *n* = 235	PTSD *n* = 44	TBI *n* = 158	PTSD with TBI *n* = 52
Age [mean (sd)]	31.78 (6.19)	32.51 (5.78)	33.52 (7.86)	33.40 (7.84)
Ethnicity [n (%)]				
Hispanic	41 (20.40%)	8 (21.62%)	41 (28.87%)	14 (32.56%)*
Non-Hispanic Asian	25 (12.44%)	4 (10.81%)	12 (8.45%)	0 (0.00%)
Non-Hispanic Black	25 (12.44%)	6 (16.22%)	10 (7.04%)	7 (16.28%)
Non-Hispanic White	102 (50.75%)	14 (37.84%)	71 (50.00%)	18 (41.86%)
Non-Hispanic Other	8 (3.98%)	5 (13.51%)	8 (5.63%)	4 (9.30%)
Education [n (%)]				
Less than 12th grade	2 (1.01%)	0 (0.00%)	3 (2.26%)	0 (0.00%)
High School Diploma or GED	46 (23.12%)	10 (27.03%)	26 (19.55%)	13 (28.89%)
2 years college, AA degree	42 (21.11%)	12 (32.43%)	39 (29.32%)	14 (31.11%)
4 years college, BA degree	84 (42.21%)	12 (32.43%)	40 (30.08%)	10 (22.22%)
Masters degree	23 (11.58%)	3 (8.11%)	23 (17.29%)	6 (13.33%)
Doctoral degree	2 (1.01%)	0 (0.00%)	2 (1.50%)	2 (4.44%)

*CLIA* Clinical Laboratory Improvement Amendments, *HC* healthy controls, *PTSD* post-traumatic stress disorder, *TBI* traumatic brain injury, *HOMA-IR* Homeostatic Model Assessment of Insulin Resistance.

*p value < 0.05.

### Discriminating PTSD cases from healthy controls

**Fig. 1:**
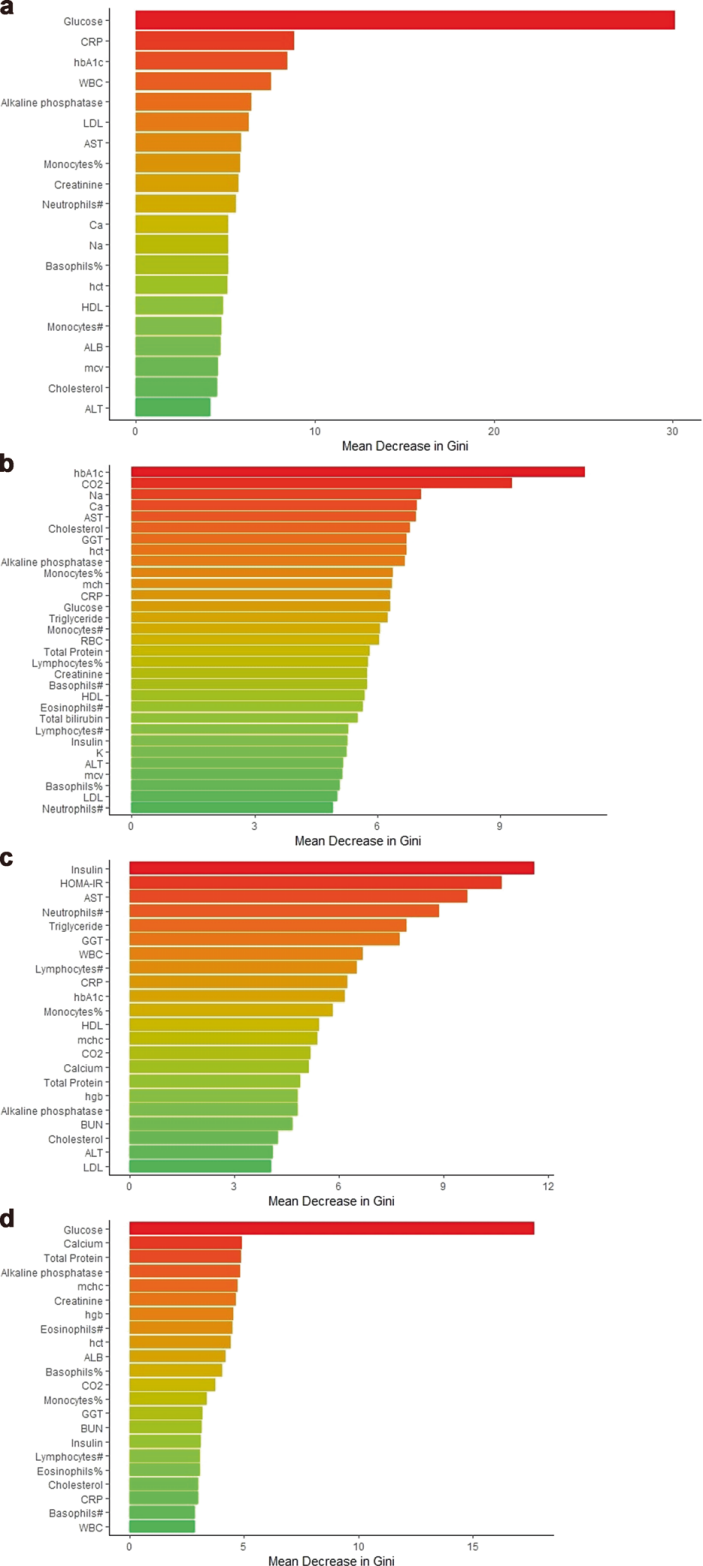
Discriminating PTSD, TBI, and PTSD comorbid with TBI from Healthy Controls- Feature Importance. **a** Discriminating PTSD from Healthy Controls- Feature Importance **b** Discriminating TBI from Healthy Controls- Feature Importance **c** Discriminating PTSD comorbid with TBI from Healthy Controls- Feature Importance **d** Discriminating PTSD from TBI- Feature Importance.

**Fig. 2:**
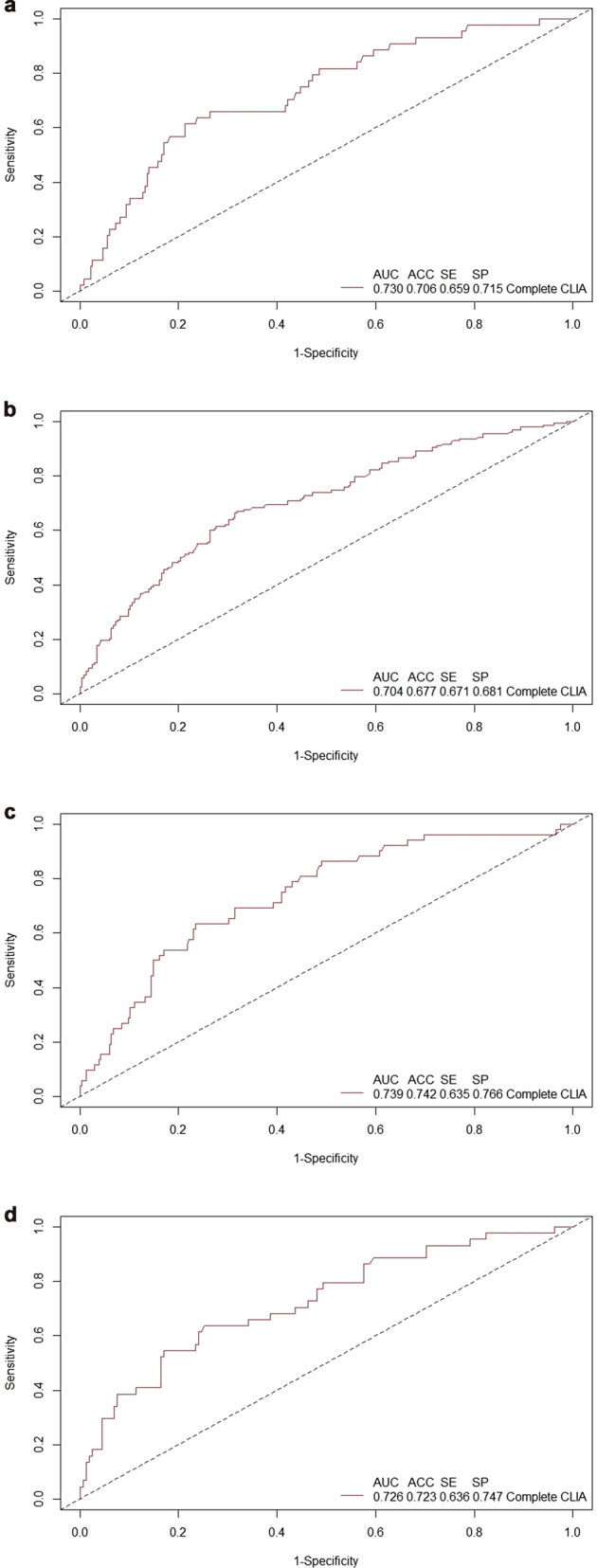
Discriminating PTSD, TBI, and PTSD comorbid with TBI from Healthy Controls- AUC, Accuracy, Sensitivity, and Specificity. **a** Discriminating PTSD from Healthy Controls - AUC, Accuracy, Sensitivity, and Specificity **b** Discriminating TBI from Healthy Controls -AUC, Accuracy, Sensitivity, and Specificity **c** Discriminating PTSD comorbid with TBI from Healthy Controls- AUC, Accuracy, Sensitivity, and Specificity **d** Discriminating PTSD from TBI- AUC, Accuracy, Sensitivity, and Specificity.

In this comparison we found glucose, CRP, hbA1c, WBC, and alkaline phosphatase as the five most important features in the RF model (Fig. 1a). The AUC, accuracy, sensitivity, and specificity using random forest with all CLIA features were 0.730, 0.706, 0.659, and 0.715, respectively (Fig. 2a). HOMA-IR was not included in the CLIA features for RF.

### Discriminating TBI from healthy controls

In this subgroup comparison, we found HbA1c, bicarbonate (CO2), sodium (Na), Calcium (Ca), and aspartate aminotransferase (AST) as the five most important features (Fig. 1b). The AUC, accuracy, sensitivity, and specificity using random forest features were 0.704, 0.677, 0.671, and 0.681, respectively (Fig. 2b). Similar to the previous subgroup comparison, HOMA-IR was not included in the CLIA features for RF.

### Discriminating PTSD with comorbid TBI from healthy controls

After including HOMA-IR in this subgroup comparison, we found insulin, HOMA-IR, AST, neutrophils count, and triglyceride as the five most important features (Fig. 1c). The AUC, accuracy, sensitivity, and specificity using random forest were 0.739, 0.742, 0.635, and 0.766, respectively (Fig. 2c).

### Discriminating PTSD from TBI

In this subgroup comparison, we found glucose, calcium (Ca), total protein, alkaline phosphatase, and mean corpuscular hemoglobin concentration (MCHC) as the five most important features (Fig. 1d). The AUC, accuracy, sensitivity, and specificity using random forest were 0.726, 0.723, 0.636, and 0.747, respectively (Fig. 2d). HOMA-IR was also included in the CLIA features for RF.

### Confounder analysis

In light of a high prevalence of comorbid BMI, depression, and alcohol abuse in PTSD and TBI patients, we investigated whether AUD, MDD or BMI were confounders in our group comparisons. Our confounder analyses revealed that none of these three features are confounders, suggesting that our Random Forest classification using CLIA blood tests classifies PTSD and TBI independently of the effects of alcohol abuse, major depressive disorder, and obesity (see Supplemental Table 1–4).

## Discussion

Developing biomarkers to screen for neuropsychiatric disorders including PTSD and mTBI is an urgent priority. A previous study from Dean et al. [9] identified 28 multi-omic blood biomarkers to accurately and reliably predict PTSD, a proof-of-concept study, which demonstrated that objective criteria are feasible. In the current study, we identified features from routine CLIA laboratory blood tests which hold promise for screening for PTSD and TBI in veterans in primary care and other medical practice settings. With independent replication implementation of this approach has potential for low cost, accessible biomarkers, permitting high throughput, nonstigmatizing screening for PTSD and TBI in military personnel and veterans in primary care and specialty medical settings. These findings are intended to advance screening, to be used in concert with PTSD and TBI diagnostic assessments based on clinical interviews and self-report measures in primary care and with the CAPS, self-report measures, neuropsychological testing and structural, functional and molecular neuroimaging in specialty mental health and neurological care. Our findings also shed light on biological mechanisms underlying the development of PTSD and TBI.

Each of the four models identified a set of CLIA features with variable importance. Of note, none of the single CLIA features results is sufficient to differentiate subjects with PTSD, mild TBI or PTSD with comorbid mild TBI from control subjects and from each other. However, using the RF technique, a combination of these CLIA features holds promise as screening measures for use in routine clinical practice. These findings are consistent with the complexity of the biology of PTSD and mTBI. They also underscore the importance of advanced statistical analysis in deciphering the multiple interactive biological features for these disorders.

The five most important biomarkers differentiating PTSD from HC from our analysis are glucose, CRP, HbA1c, WBC, and alkaline phosphatase. Each feature, except alkaline phosphatase, is greater in the PTSD than the HC group. Elevated glucose and HbA1c are indicative of impaired glucose metabolism, which may eventually lead to pre-diabetes and diabetes. Elevated CRP and WBC levels are associated with an inflammatory state. Together, the elevation of these four markers in the PTSD group is consistent with clinical signs of pre-diabetes and systematic inflammation. Given these findings we would expect that our PTSD subjects would have elevated AST and ALT, markers for subclinical impaired liver function. This is the case, even though these two markers are not the most important features identified in RF. Interestingly, we found that compared to HC alkaline phosphatase levels are lower in the PTSD group, instead of being increased as occurs in impaired liver function. Decreased alkaline phosphatase, which is relatively rare, is sometimes associated with mineral deficiency, such as zinc and magnesium. It is possible that alkaline phosphatase becomes elevated later in impaired liver function. von Känel and colleagues found that PTSD symptom severity following myocardial infarction was associated with higher levels of alkaline phosphatase [19]. Alternatively, PTSD subjects may have mineral deficiency which has not been previously reported. At this point, the relevance of decreased alkaline phosphatase in our PTSD subjects requires further clarification.

In our classification models differentiating mTBI and HC, the five most prominent features are HbA1c, CO2, Na, Ca, and AST. Except for CO2, these markers have increased values in the mTBI group. Similar to our PTSD group, elevated HbA1c is associated with impaired glucose metabolism. Elevation of AST is a sign of impaired liver function, which can occur with systematic inflammation, insulin resistance, or excessive alcohol intake. As expected, ALT is also elevated in the mTBI subjects, even though ALT is not among the most important features identified in RF. The other three features are electrolytes, which can be dysregulated in severe TBI patients due to neuroendocrine and metabolic changes. For example, hypernatremia is commonly associated with TBI with multiple mechanisms, including postconcussive diabetes insipidus from impaired ADH secretion, water loss from diuretics, and hypertonic sodium intake [20]. On the other hand, hypercalcemia in TBI patients may be associated with immobilization or neuroendocrine influence in bone remodeling [21]. Decreased serum CO2 can be associated with respiratory and metabolic acidosis, which can result from a variety of causes, such as chronic lung or kidney disease.

In our RF model for discriminating PTSD comorbid with TBI from HC, the five most prominent features are insulin, HOMA-IR, AST, neutrophils count, and triglyceride. All five features are elevated in PTSD comorbid with TBI. Insulin, similar to HOMA-IR, is another marker for glucose metabolism. Elevated neutrophil count is a sign of inflammation. Elevated triglyceride, similar to elevated AST, can be associated with metabolic syndrome, diabetes, or excessive alcohol intake.

Interestingly, electrolytes are not among the most prominent features. This could result from the more severe abnormalities in glucose metabolism and inflammation, which can mask the relatively subtle changes in electrolytes. These results are not surprising as there was elevation in HDL, HbA1c levels, and CRP levels, which suggest that subjects with TBI and PTSD have already developed sub-syndromal clinical signs of metabolic syndrome, although the level of abnormality has not reached a diagnostic threshold.

Our model for discriminating between PTSD and mTBI identified glucose, Ca, total protein, alkaline phosphatase, and MCHC as the most prominent features. Except for MCHC, all of these features are increased in the PTSD group relative to the mTBI group. Once again, glucose is among the most prominent features, suggesting that impaired glucose metabolism is a more important feature for PTSD than mTBI. Elevated alkaline phosphatase is suggestive of impaired liver function. While elevated total protein is associated with dehydration, decreased MCHC may suggest anemia. Both PTSD and mTBI subjects have higher levels of calcium compared to HC. The higher levels of calcium in PTSD than in mTBI may be explained by greater reductions in mobility due to social withdrawal. Alternatively, differences in calcium levels may be explained by different neuroendocrine changes in PTSD from mTBI with an unclear clinical significance.

In overview our study showed that in our RF models, routine clinical blood tests for assessing glucose metabolism and inflammatory processes are often among the most prominent features (glucose, insulin, HOMA-IR, HbA1c, CRP). RF models test for complex higher order interactions among all of the variables in the model. Identification of these features suggests that metabolic disturbances with impaired glucose tolerance and insulin resistance could be an important biological characteristic of PTSD. Importantly, dysregulation of glucose metabolism likely precedes the development of other clinical abnormalities such as systematic inflammation, dyslipidemia, and cardiovascular diseases. As impaired glucose metabolism is a significant risk factor for cardiovascular disease, these results also underscore the importance of using advanced statistical models to make clinical predictions based on subthreshold diagnostic laboratory values. These findings are in line with previous reports of US veterans indicating PTSD and other mental illnesses increase cardiovascular risks [22].

PTSD may increase the risk of insulin resistance and diabetes through several possible mechanisms. It is associated with elevated inflammatory markers, such as C-reactive protein, interleukin 6 (IL-6) and tumor necrosis factor (TNF), which can increase insulin resistance [19, 23]. It is also associated with alterations to the hypothalamic-pituitary-adrenal axis, often with paradoxically low cortisol [24, 25], which has been shown linked to visceral obesity and insulin resistance. PTSD is also associated with elevated BMI, poor sleep, and lifestyle changes including sedentary behavior and poor dietary choices, which all contribute to the development of diabetes [26, 27].

Our study has identified several CLIA features which hold promise for differentiating TBI and TBI with PTSD from healthy control subjects. Subgroup comparisons between HC and TBI showed that biomarkers in glucose metabolism (HbA1c) are among the most important features, which suggests that glucose intolerance and insulin resistance can also be a part of the pathophysiology of TBI.

Systematic inflammation can be another aspect of the pathophysiology of TBI. Neuronal injury from TBI can trigger an inflammatory molecular cascade in the area surrounding the injury, stimulating the release of proinflammatory cytokines from glial cells, as well as deregulating angiogenic factors and activating metalloproteases. These events are likely to cause damage and leakage of the blood-brain barrier and may lead to systemic inflammation, with changes in percentage of different peripheral WBCs, including lymphocytes [28, 29]. Indeed, systemic inflammation has been hypothesized as a consequence of both TBI and PTSD [30]. This systemic inflammation hypothesis is also confirmed by the concomitant increase in CRP, a well-known biomarker of inflammatory responses which is synthetized by the liver following interleukin-6 secretion by macrophages and T cells.

Biomarkers in lipid metabolism are also often among the most prominent features, although they are not among the top five features in all of the group comparisons. A disturbance in lipid metabolism was observed in the brain of a TBI mouse model [31]. Lower levels of several major phospholipid (PL) classes have been reported in TBI, PTSD, and TBI with PTSD groups, compared with healthy controls [32]. Lower PL levels have been reported in those with moderate-to-severe PTSD. In a clinical study, monounsaturated fatty acid–containing phosphatidylcholine (PC) and phosphatidylinositol (PI) species were lower in the TBI and TBI with PTSD groups [32].

## Conclusion

PTSD and mild TBI have overlapping symptoms, with few previously reported abnormalities in routine clinical laboratory and imaging findings, presenting diagnostic challenges. Our study has shown that with advanced statistical techniques using random forest methods, we were able to utilize routine CLIA laboratory blood tests to differentiate PTSD and mild TBI from healthy controls and from each other. Our results have further shown that increased glucose and insulin resistance levels and upregulation of inflammatory markers are promising blood biomarkers for low cost, high-throughput diagnostic screening for both PTSD and mild TBI. Independent validation of our results in new samples of veterans and service members are required to support the implementation of CLIA labs for screening for military service-related PTSD and TBI in medical practice settings.

## Limitations

There are a several limitations to this study. We do not propose our blood biomarkers as definitive diagnostic tests, rather as low cost, high throughput widely accessible initial screening measures to be used in conjunction with patient self-reports, clinical interviews and where feasible specialized neuropsychological testing and neuroimaging procedures. Additionally, the number of participants in the PTSD and mTBI groups is smaller than the HC group, which biases the model to predict the majority class in all cases. To address this issue, SMOTE was used to rebalance our data. Furthermore, the accuracies of the models are less than ideal for screening measures, necessitating refinement in down selection of the features in future studies.

The consistent ability of CLIA labs to discriminate between the psychopathology group contrasts provide some validation evidence of CLIA labs ability to separate PTSD cases from controls. Importantly, while the random forest OOB algorithm provides internal validation for all classification contrasts made, external validation is still required. In exploratory analyses we attempted to replicate our findings utilizing CLIA labs to differentiate PTSD cases and healthy controls in the CVC cohort in an independent data set of OEF and OIF veterans, designated the Systems Biology Cohort (SBC) (Dean and colleagues, 2020). The SBC comprises 83 PTSD cases and 82 combat exposed healthy controls. We did not recruit for an mTBI group in the SBC cohort. Applying the CLIA lab model from our discovery analyses in the CVC Cohort, we found lower overall accuracy in the replication sample, 0.706 in the CVC discovery sample versus 0.606 in the SBC external validation sample, with the AUC declining from 0.73 to 0.60. This reduced accuracy may be attributable in part to sample size limitations and heterogeneity of clinical and CLIA lab features in the CVC and SBC cohorts. Given these limitations, replication of our findings in an independently recruited larger cohort of veterans with CLIA labs ascertained in PTSD, mTBI, and warzone-deployed healthy controls, with more balanced numbers in each group, is needed as a next step towards validating our findings.

## Supplementary information

Supplemental Material

## Data Availability

An R-code implementing the considered tests in our paper is available with this paper at C.R.M’s website at New York University Langone Health.
