# Supplementary material for: Screening for PTSD and TBI in Veterans using Routine Clinical Laboratory Blood Tests
Source: Transl Psychiatry. 2023 Feb 21;13:64. doi: 10.1038/s41398-022-02298-x (PMC9944218; doi:10.1038/s41398-022-02298-x)
Supplement: Supplementary file 1 — Supplemental Material [file 41398_2022_2298_MOESM1_ESM.docx]

**Supplemental Table 1: Confounder Analysis – HC vs PTSD: Results of significance tests of confounder hypotheses for AUD, current depression (MDD), and BMI**

|  | AUD | | MDD | | BMI | |
| --- | --- | --- | --- | --- | --- | --- |
| Features | Test: P(F\|AUD)=P(F) | Test: P(PTSD\|F) =P(PTSD\|F,AUD) | Test: P(F\|MDD)=P(F) | Test: P(PTSD\|F) =P(PTSD\|F,MDD) | Test: P(F\|BMI)=P(F) | Test: P(PTSD\|F) =P(PTSD\|F,BMI) |
| hbA1c | 1.000 | **0.013** | 1.000 | **0.005** | **0.001** | 0.771 |
| glucose | 1.000 | **<0.001** | 1.000 | **<0.001** | **<0.001** | 0.309 |
| Na | 1.000 | 0.464 | 1.000 | 0.918 | 0.527 | 0.549 |
| Ca | 1.000 | 0.181 | 1.000 | **0.001** | 0.361 | 0.457 |
| ALB | 1.000 | 0.271 | 1.000 | 0.745 | 0.543 | 0.563 |
| Alkaline phosphatase | 1.000 | **<0.001** | 1.000 | **<0.001** | **0.019** | 0.645 |
| AST | 1.000 | **<0.001** | 1.000 | **<0.001** | **<0.001** | 0.533 |
| ALT | 1.000 | **<0.001** | 1.000 | **<0.001** | **<0.001** | 0.120 |
| creatinine | 1.000 | 0.032 | 1.000 | 0.070 | 0.300 | 0.837 |
| Cholesterol | 1.000 | **<0.001** | 1.000 | **<0.001** | **0.007** | 0.863 |
| HDL | 1.000 | **<0.001** | 1.000 | **<0.001** | **<0.001** | 0.216 |
| LDL | 1.000 | **<0.001** | 1.000 | **<0.001** | **<0.001** | 0.890 |
| WBC | 1.000 | **0.002** | 1.000 | **<0.001** | **0.015** | 0.358 |
| hct | 1.000 | **<0.001** | 1.000 | **<0.001** | 0.063 | 0.675 |
| mcv | 1.000 | **<0.001** | 1.000 | **<0.001** | 0.099 | 0.267 |
| Monocytes% | 1.000 | 0.511 | 1.000 | 0.181 | 0.481 | 0.707 |
| Basophils% | 1.000 | 0.061 | 1.000 | 0.203 | 1.000 | **0.013** |
| Neutrophils# | 1.000 | **0.001** | 1.000 | **<0.001** | **0.049** | 0.384 |
| Monocytes# | 1.000 | 0.089 | 1.000 | 0.777 | **0.028** | 0.621 |
| CRP | 1.000 | 0.099 | 1.000 | **<0.001** | **<0.001** | 0.343 |

*HC* healthy controls, *PTSD* post-traumatic stress disorder, *AUD* alcohol use disorder, *BMI* body mass index

Bold: p<.05

P(F) = Probability of feature F

P(F|AUD) = Probability of feature given AUD

P(PTSD|F, AUD) = Probability of PTSD given feature and AUD

P(F|MDD) = Probability of feature given MDD

P(PTSD|F, MDD) = Probability of PTSD given feature and MDD

P(F|BMI) = Probability of feature given BMI

P(PTSD|F, BMI) = Probability of PTSD given feature and BMI

**Supplemental Table 2: Confounder Analysis – HC vs TBI: Results of significance tests of confounder hypotheses for AUD, current depression (MDD), and BMI**

|  | AUD | | MDD | | BMI | |
| --- | --- | --- | --- | --- | --- | --- |
| Features | Test: P(F\|AUD)=P(F) | Test: P(TBI\|F) =P(TBI\|F,AUD) | Test: P(F\|MDD)=P(F) | Test: P(TBI\|F) =P(TBI\|F,MDD) | Test: P(F\|BMI)=P(F) | Test: P(TBI\|F) =P(TBI\|F,BMI) |
| hbA1c | 1.000 | **<0.001** | 1.000 | **<0.001** | **<0.001** | 0.932 |
| glucose | 1.000 | **<0.001** | 1.000 | **<0.001** | **<0.001** | 0.849 |
| Na | 1.000 | **<0.001** | 1.000 | **<0.001** | 0.065 | 0.893 |
| K | 1.000 | **<0.001** | 1.000 | **<0.001** | 0.772 | 0.958 |
| CO2 | 1.000 | **<0.001** | 1.000 | **<0.001** | 0.446 | 0.870 |
| Ca | 1.000 | **<0.001** | 1.000 | **<0.001** | 0.231 | 0.837 |
| Total Protein | 1.000 | **<0.001** | 1.000 | **<0.001** | 0.617 | 0.962 |
| Total bilirubin | 1.000 | **<0.001** | 1.000 | **<0.001** | 1.000 | 0.631 |
| Alkaline phosphatase | 1.000 | **<0.001** | 1.000 | **<0.001** | **<0.001** | 0.824 |
| AST | 0.801 | **<0.001** | 1.000 | **<0.001** | **<0.001** | 0.975 |
| ALT | 1.000 | **<0.001** | 1.000 | **<0.001** | **<0.001** | 0.960 |
| GGT | 1.000 | **<0.001** | 1.000 | **<0.001** | **<0.001** | 0.545 |
| creatinine | 1.000 | **<0.001** | 1.000 | **<0.001** | **0.029** | 0.745 |
| Cholesterol | 1.000 | **<0.001** | 1.000 | **<0.001** | **0.008** | 0.975 |
| Triglyceride | 1.000 | **<0.001** | 1.000 | **<0.001** | **<0.001** | 0.938 |
| HDL | 1.000 | **<0.001** | 1.000 | **<0.001** | **<0.001** | 0.990 |
| LDLD | 1.000 | **<0.001** | 1.000 | **<0.001** | **<0.001** | 0.989 |
| RBC | 0.993 | **<0.001** | 1.000 | **<0.001** | **<0.001** | 0.824 |
| hct | 1.000 | **<0.001** | 1.000 | **<0.001** | **0.010** | 0.886 |
| mcv | 1.000 | **<0.001** | 1.000 | **<0.001** | **<0.001** | 0.871 |
| mch | 1.000 | **<0.001** | 1.000 | **<0.001** | **0.004** | 0.852 |
| Lymphocytes% | 1.000 | **<0.001** | 1.000 | **<0.001** | 0.563 | 0.935 |
| Monocytes% | 1.000 | **<0.001** | 1.000 | **<0.001** | 0.155 | 0.952 |
| Basophils% | 1.000 | **<0.001** | 1.000 | **<0.001** | 1.000 | 0.084 |
| Neutrophils# | 1.000 | **<0.001** | 1.000 | **<0.001** | **0.018** | 0.888 |
| Lymphocytes# | 1.000 | **<0.001** | 1.000 | **<0.001** | **0.036** | 0.877 |
| Monocytes# | 1.000 | **<0.001** | 1.000 | **<0.001** | **0.004** | 0.879 |
| Eosinophils# | 1.000 | **<0.001** | 1.000 | **<0.001** | **0.009** | 0.892 |
| Basophils# | 1.000 | **<0.001** | 1.000 | **<0.001** | 0.647 | 0.962 |
| insulin | 1.000 | **<0.001** | 1.000 | **<0.001** | **<0.001** | 0.607 |
| CRP | 1.000 | **<0.001** | 1.000 | **<0.001** | **<0.001** | 0.883 |

*HC* healthy controls, *PTSD* post-traumatic stress disorder, *TBI* traumatic brain injury, *AUD* alcohol use disorder, *BMI* body mass index

Bold: p<.05

P(F) = Probability of feature F

P(F|AUD) = Probability of feature given AUD

P(TBI|F, AUD) = Probability of TBI given feature and AUD

P(F|MDD) = Probability of feature given MDD

P(TBI|F, MDD) = Probability of TBI given feature and MDD

P(F|BMI) = Probability of feature given BMI

P(TBI|F, BMI) = Probability of TBI given feature and BMI

**Supplemental Table 3: Confounder Analysis – HC vs PTSD with TBI: Results of significance tests of confounder hypotheses for AUD, current depression (MDD), and BMI**

|  | AUD | | MDD | | BMI | |
| --- | --- | --- | --- | --- | --- | --- |
| Features | Test: P(F\|AUD)=P(F) | Test: P(PTSD-TBI\|F) =P(PTSD\|F,AUD) | Test: P(F\|MDD)=P(F) | Test: P(PTSD-TBI\|F) =P(PTSD\|F,MDD) | Test: P(F\|BMI)=P(F) | Test: P(PTSD-TBI\|F) =P(PTSD\|F,BMI) |
| hbA1c | 1.000 | 0.395 | 1.000 | 0.086 | 0.002 | 0.878 |
| BUN | 1.000 | 0.016 | 1.000 | 0.039 | 0.876 | 0.409 |
| CO2 | 1.000 | 0.018 | 1.000 | 0.880 | 0.500 | 0.145 |
| Ca | 1.000 | 0.420 | 1.000 | 0.009 | 0.499 | 0.782 |
| Total Protein | 1.000 | 0.010 | 1.000 | <0.001 | 0.411 | 0.929 |
| Alkaline phosphatase | 1.000 | <0.001 | 1.000 | <0.001 | 0.002 | 0.995 |
| AST | 1.000 | <0.001 | 1.000 | <0.001 | <0.001 | 0.935 |
| ALT | 1.000 | <0.001 | 1.000 | <0.001 | <0.001 | 0.839 |
| GGT | 1.000 | <0.001 | 1.000 | <0.001 | <0.001 | 0.508 |
| Cholesterol | 1.000 | <0.001 | 1.000 | <0.001 | <0.001 | 0.652 |
| Triglyceride | 1.000 | <0.001 | 1.000 | <0.001 | <0.001 | 0.969 |
| HDL | 1.000 | <0.001 | 1.000 | <0.001 | <0.001 | 0.967 |
| LDL | 1.000 | <0.001 | 1.000 | <0.001 | <0.001 | 0.735 |
| WBC | 1.000 | <0.001 | 1.000 | <0.001 | 0.015 | 0.898 |
| hgb | 1.000 | <0.001 | 1.000 | <0.001 | 0.158 | 0.552 |
| mchc | 1.000 | <0.001 | 1.000 | <0.001 | 0.716 | 0.646 |
| Monocytes% | 1.000 | 0.002 | 1.000 | 0.002 | 0.661 | 0.343 |
| Neutrophils# | 1.000 | <0.001 | 1.000 | <0.001 | 0.131 | 0.544 |
| Lymphocytes# | 1.000 | <0.001 | 1.000 | <0.001 | 0.009 | 0.930 |
| insulin | 1.000 | 0.750 | 1.000 | 0.058 | <0.001 | 0.736 |
| CRP | 1.000 | <0.001 | 1.000 | <0.001 | <0.001 | 0.687 |
| HOMA-IR | 1.000 | <0.001 | 1.000 | <0.001 | <0.001 | 0.542 |

*HC* healthy controls, *PTSD* post-traumatic stress disorder, *TBI* traumatic brain injury, *AUD* alcohol use disorder, *BMI* body mass index

P(F) = Probability of feature F

P(F|AUD) = Probability of feature given AUD

P(PTSD-TBI|F, AUD) = Probability of PTSD with TBI given feature and AUD

P(F|MDD) = Probability of feature given MDD

P(PTSD-TBI|F, MDD) = Probability of PTSD with TBI given feature and MDD

P(F|BMI) = Probability of feature given BMI

P(PTSD-TBI|F, BMI) = Probability of PTSD with TBI given feature and BMI

**Supplemental Table 4: Confounder Analysis –TBI vs PTSD: Results of significance tests of confounder hypotheses for AUD, current depression (MDD), and BMI**

|  | AUD | | MDD | | BMI | |
| --- | --- | --- | --- | --- | --- | --- |
| Features | Test: P(F\|AUD)=P(F) | Test: P(PTSD\|F) =P(PTSD\|F,AUD) | Test: P(F\|MDD)=P(F) | Test: P(PTSD\|F) =P(PTSD\|F,MDD) | Test: P(F\|BMI)=P(F) | Test: P(PTSD\|F) =P(PTSD\|F,BMI) |
| glucose | 1.000 | <0.001 | 1.000 | <0.001 | **0.038** | 0.545 |
| BUN | 1.000 | 0.959 | 1.000 | 0.041 | 0.788 | 0.892 |
| CO2 | 1.000 | 0.323 | 1.000 | <0.001 | 0.187 | 0.970 |
| Ca | 1.000 | 0.183 | 1.000 | 0.029 | 0.089 | 0.804 |
| Total Protein | 1.000 | 0.027 | 0.955 | 0.012 | 0.399 | 0.988 |
| ALB | 1.000 | 0.209 | 1.000 | 0.427 | 0.124 | 0.989 |
| Alkaline phosphatase | 1.000 | 0.001 | 1.000 | <0.001 | **0.004** | 0.461 |
| GGT | 0.949 | <0.001 | 1.000 | <0.001 | **<0.001** | 0.933 |
| creatinine | 1.000 | 0.001 | 0.447 | 0.003 | 0.091 | 0.987 |
| Cholesterol | 0.988 | <0.001 | 1.000 | 0.002 | 0.770 | 0.796 |
| WBC | 1.000 | <0.001 | 1.000 | 0.004 | **0.005** | 0.650 |
| hgb | 1.000 | 0.015 | 0.634 | 0.005 | **0.048** | 0.981 |
| hct | 1.000 | 0.005 | 0.871 | 0.003 | 0.107 | 0.996 |
| mchc | 1.000 | <0.001 | 1.000 | 0.023 | 0.910 | 0.989 |
| Monocytes% | 1.000 | 0.136 | 1.000 | 0.472 | 0.137 | 0.962 |
| Eosinophils% | 1.000 | <0.001 | 1.000 | 0.049 | 1.000 | 0.804 |
| Basophils% | 1.000 | 0.010 | 1.000 | 0.220 | 0.452 | 0.938 |
| Lymphocytes# | 1.000 | 0.001 | 1.000 | 0.001 | 0.482 | 0.803 |
| Eosinophils# | 1.000 | 0.001 | 1.000 | 0.004 | 0.395 | 0.913 |
| Basophils# | 1.000 | 0.004 | 1.000 | 0.021 | 0.604 | 0.643 |
| insulin | 1.000 | 0.025 | 1.000 | 0.429 | **<0.001** | 0.971 |
| CRP | 1.000 | 0.536 | 1.000 | 0.452 | **<0.001** | 0.913 |

*HC* healthy controls, *PTSD* post-traumatic stress disorder, *TBI* traumatic brain injury, *AUD* alcohol use disorder, *BMI* body mass index

P(F) = Probability of feature F

P(F|AUD) = Probability of feature given AUD

P(PTSD|F, AUD) = Probability of PTSD given feature and AUD

P(F|MDD) = Probability of feature given MDD

P(PTSD|F, MDD) = Probability of PTSD given feature and MDD

P(F|BMI) = Probability of feature given BMI

P(PTSD|F, BMI) = Probability of PTSD given feature and BMI

**Supplemental Figure 1: Boxplot – HC vs PTSD**


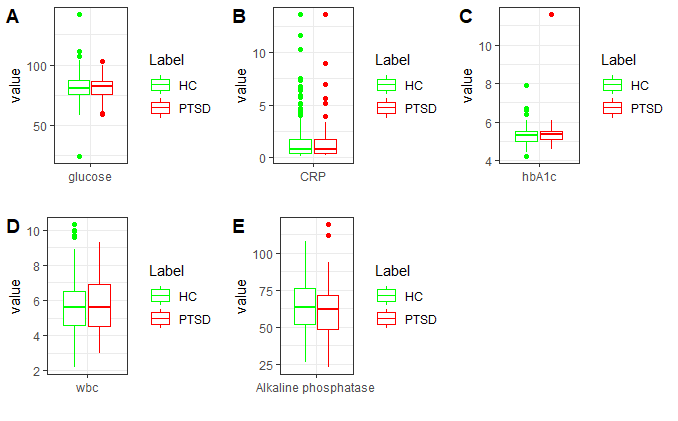


**Supplemental Figure 2: Boxplot – HC vs TBI**

**
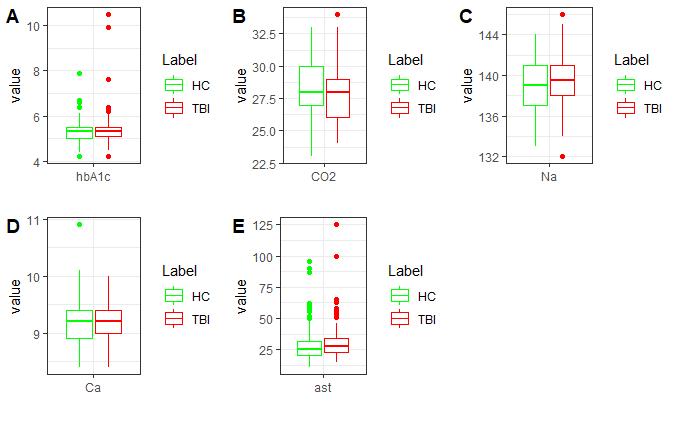
**

**Supplemental Figure 3: Boxplot – HC vs PTSD with TBI**

**
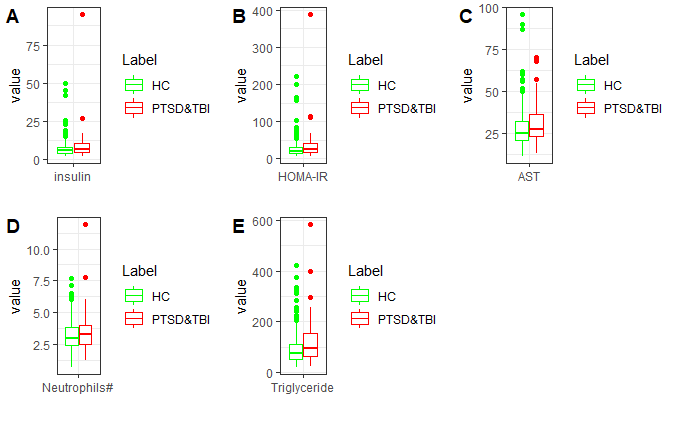
**

**Supplemental Figure 4: Boxplot – TBI vs PTSD**

**
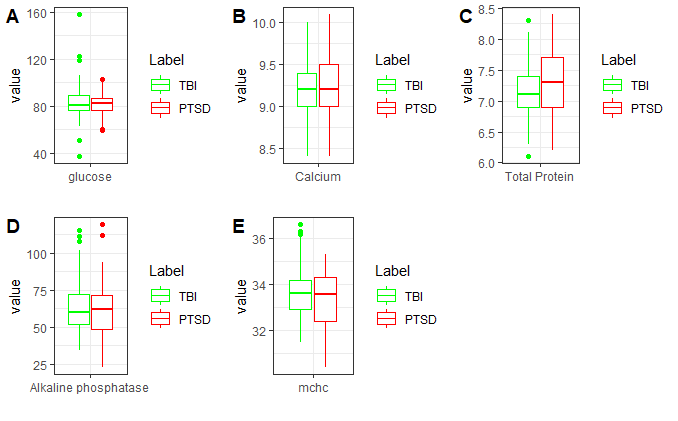
**
